# A multicentre, open-label, single-arm phase II trial of the efficacy and safety of sclerotherapy using 3% polidocanol foam to treat second-degree haemorrhoids (SCLEROFOAM)

**DOI:** 10.1007/s10151-022-02609-w

**Published:** 2022-03-25

**Authors:** G. Gallo, R. Pietroletti, E. Novelli, A. Sturiale, R. Tutino, P. Lobascio, R. Laforgia, E. Moggia, M. Pozzo, M. Roveroni, V. Bianco, A. Realis Luc, A. Giuliani, E. Diaco, G. Naldini, M. Trompetto, R. Perinotti, G. Sammarco

**Affiliations:** 1 Department of Medical and Surgical Sciences, University of Catanzaro, Catanzaro, Italy; 2 Proctology Unit, Department of Biotechnological and Applied Clinical Sciences, University of L’Aquila, L’Aquila, Italy; 3 Biostat Research S.a.S, Borgomanero, Italy; 4 Proctology and Pelvic Floor Clinical Centre, Cisanello University Hospital, Pisa, Italy; 5 Chirurgia 1, Azienda ULSS 2 Marca Trevigiana, Ospedale Regionale Treviso, Treviso, Italy; 6 Surgical Unit “M. Rubino” Department of Emergency and Organ Transplantation, University Aldo Moro of Bari, Bari, Italy; 7 Department of General Surgery, Infermi Hospital, Rivoli, Torino, Italy; 8 Department of General Surgery, “Degli Infermi” Hospital, Biella, Italy; 9 Department of Surgery, Aosta Hospital, Aosta, Italy; 10 General Surgery Unit, Cetraro Hospital, Cetraro, Italy; 11 Department of Colorectal Surgery, S. Rita Clinic, Vercelli, Italy; 12 General Surgery Unit, Department of Biotechnological and Applied Clinical Sciences, San Salvatore Hospital, University of L’Aquila, L’Aquila, Italy; 13 Minerva Surgical Service, Catanzaro, Italy; 14 Department of Health Sciences, University of Catanzaro, Catanzaro, Italy; 15 Department of Medicine, Surgery and Neurosciences, Unit of General Surgery and Surgical Oncology, University of Siena, Siena, Italy

**Keywords:** Haemorrhoidal disease, Polidocanol foam, Safety, Sclerotherapy, Bleeding haemorrhoids

## Abstract

**Background:**

The aim of the present study was to evaluate the efficacy and safety of 3% polidocanol foam for treating 2nd-degree haemorrhoids.

**Methods:**

A multicentre, open-label, single-arm, phase 2 trial involving 10 tertiary referral centres for haemorrhodal disease (HD) was performed. Between January and June 2019, patients with 2nd-degree haemorrhoids were prospectively included in this study. The primary outcome was to establish the success rate after one sclerotherapy session in terms of complete resolution of bleeding episodes one week after the injection. The Hemorrhoidal Disease Symptom Score (HDSS), the Short Health Scale for HD (SHS-HD) score and the Vaizey incontinence score were used to assess symptoms and their impact on quality of life and continence. Pain after the procedure, subjective symptoms and the amount and type of painkillers used were recorded. Patients were followed up for 1 year.

**Results:**

There were 183 patients [111 males; 60.7%, mean age 51.3 ± 13.5 (18–75) years]. Complete resolution of bleeding was reached in 125/183 patients (68.3%) at 1 week and the recurrence rate was 12% (15/125).

Thirteen patients (7.4%) underwent a second sclerotherapy session, while only 1 patient (1.8%) had to undergo a third session. The overall 1-year success rate was 95.6% (175/183). The HDSS and the SHS score significantly improved from a median preoperative value of 11 and 18 to 0 and 0, respectively (*p *< 0.001). There were 3 episodes of external thrombosis. No serious adverse events occurred.

**Conclusions:**

Sclerotherapy with 3% polidocanol foam is a safe, effective, painless, repeatable and low-cost procedure in patients with bleeding haemorrhoids.

## Introduction

Haemorrhoidal disease (HD) is one of the most frequent proctological diseases, even if its true prevalence in the population is unknown because patients are often too embarrassed to schedule an appropriate specialist visit [1].

Therefore, to reduce the symptoms as well as patient discomfort, the pharmacological and technological research on HD is constantly evolving and Cosman’s description of the “piles of money” associated with the industry of diagnosing and treating HD is becoming increasingly appropriate [2].

The treatment of HD has been standardized according to Goligher's classification even if the latter refers only to mechanical prolapse and does not consider etiopathogenesis, patient symptoms and quality of life and special conditions such as pregnancy, coagulopathies, inflammatory bowel disease (IBD), immunosuppression or age ≥ 65. In addition, some clinical scenarios, such as circumferential prolapse or single piles that require a tailored treatment, are not considered [3].

Consequently, the development of tailored surgeries has revolutionized the treatment of HD by focusing more attention on the symptoms of HD [4]; this development went hand in hand with the publication and validation of new scoring systems for assessing patient outcomes [5].

In this context, sclerotherapy, usually recommended for 1st–2nd-degree haemorrhoids or patients with 3^rd^-degree haemorrhoids who failed conservative treatment [3], plays an important role in the symptomatic treatment of bleeding haemorrhoids, with indications in elderly or comorbid patients who cannot undergo traditional surgical treatment [6]. Moreover, even without a sufficient degree of evidence, it seems that the use of foam has reduced postoperative complications [7].

Currently, 3% polidocanol (Aethoxysklerol^®^ 3%, Chemische Fabrik Kreussler & Co. GmbH, Wiesbaden, Germany) is the most commonly used and approved sclerosing agent in Italy for treating 1st- and 2nd-degree haemorrhoids.

The aim of this multicentre, open-label, single-arm phase II trial was to evaluate the efficacy and safety of 3% polidocanol foam for treating 2nd-degree haemorrhoids.

## Materials and methods

### Study design

This was a multicentre, open label, single-arm, phase 2 trial involving 10 tertiary referral centres for HD in Italy that were selected by the Italian Society of Colorectal Surgery [Società Italiana di Chirurgia Colorettale, (SICCR)] to assess the efficacy and safety of 3% polidocanol foam.

Demographic data, the degree of symptoms of HD, quality of life and continence level and operative details were collected through prospective data collection. Monthly data monitoring at the participating centres was performed. The protocol was approved by all ethics committees at all study centres and registered with ClinicalTrials.gov, NCT03791775. The study was conducted in accordance with the Declaration of Helsinki (1996) and International Conference on Harmonization-Good Clinical Practice (ICH-GCP) guidelines.

After enrolment (baseline, T0), patients were followed up at 1 week (T1), 4 weeks (T2), 3 months (T3), 6 months (T4) and 12 months (T5) after the procedure. The treatment was administered at T0 after baseline evaluation of the inclusion and exclusion criteria.

Written informed consent was obtained from all the patients included in the study.

A proctological examination with anoscopy was performed in all patients prior to the procedure to confirm the extent of HD and rule out any associated anorectal diseases.

The follow-up consisted of external clinical evaluation at T1 and a complete proctological evaluation, including digital rectal examination and anoscopy, from T2 to T5.

Bleeding was investigated using the Giamundo score (0 = absence of bleeding, 1 =  < 1 episode per month, 2 = 1 episode per week, 3 = 1–3 episodes per week and 4 = 4 or more episodes per week) at T0 and all follow-up visits [8].

Symptom severity and quality of life were assessed using the Hemorrhoidal Disease Symptom Score (HDSS), a 5-item questionnaire evaluating pain, itching, bleeding, soiling and prolapse on a 5-point scale (0 = never, 1 = less than once a month, 2 = less than once a week, 3 = 1–6 days per week, 4 = every day or always) and the Short Health Scale for HD (SHS-HD) score, which includes 4 questions with a 7-point Likert scale for each question (1 = minimum score, 7 = maximum score), at T0, T3 and T5 [9].

The Vaizey incontinence score (minimum score = 0, perfect continence/maximum score = 24, totally incontinent) was used to evaluate anal continence at T0, T2 and T5 [10].

Procedural pain was assessed with a visual analogue scale (VAS) score (minimum score = 0, maximum score = 10). The amount (0 = no painkillers needed; 1 = 1–3 per day for less than 2 days; 2 = 1–3 per day for more than 2 days; 3 = more than 3 per day for more than 2 days) and type (0 = no painkillers needed; 1 = minor analgesics: 1 g paracetamol; 2 = major analgesics: 600 mg ibuprofen; paracetamol and codeine) of painkillers and resumption of normal activities (0 = immediate and/or within 1 day; 1 = after 1 day and before 2 days; 2 = after 2 days and before 3 days; 3 = after more than 3 days and within 1 week), including work, were recorded from T0 to T5.

A clinical diary was delivered to the patient for the subjective assessment of itching (0 = none; 1 = sporadic; 2 = during defecation; 3 = persistent), soiling (0 = none; 1 = sporadic; 2 = persistent) and tenesmus (0 = none; 1 = sporadic; 2 = persistent) at all study visits.

Successful treatment was defined as the complete absence of bleeding episodes 1 week after the procedure based on the Giamundo bleeding score [8].

Recurrences were defined as the new onset of bleeding after T1 in the successfully treated patients, namely, from a bleeding score of 0 to at least 2 at any time point between T2 and T5.

### Eligibility criteria

Patients aged between 18 and 75 years with symptomatic 2nd-degree haemorrhoids, who failed conservative treatment, according to the Goligher classification [11] were eligible for the study.

The exclusion criteria were as follows: a history of cardiac disease, coagulopathy and anticoagulant therapies, colorectal or anal neoplasia, IBD, other proctological diseases (anal fistulas and fissures; thrombosed internal or external haemorrhoids), previous anal surgical procedures, previous sclerotherapy or rubber band ligation in the last 12 months, positive pregnancy test and breastfeeding, hepatitis B virus hepatitis C virus, or human immunodeficiency virus infection, proctitis, known allergy to polidocanol, or pelvic radiotherapy. The inability to return for postoperative follow-up visits was also considered an exclusion criterion.

### Procedure (treatment plan)

Polidocanol (Lauromacrogol 400 (INN), H3C-(CH2)11-(O-CH2-CH2)n∼9-OH) is a non-ionic detergent that mainly targets endothelial cells, consisting of a fatty alcohol part containing 12 carbon atoms and a chain of several oxyethylene units (–O–CH2–CH2) that are connected via ether (–O–) bonds. The number of oxyethylene units is between 1 and 24 and the average number of oxyethylene units is 9, which is expressed by *N *= 9 [7].

**Fig. 1:**
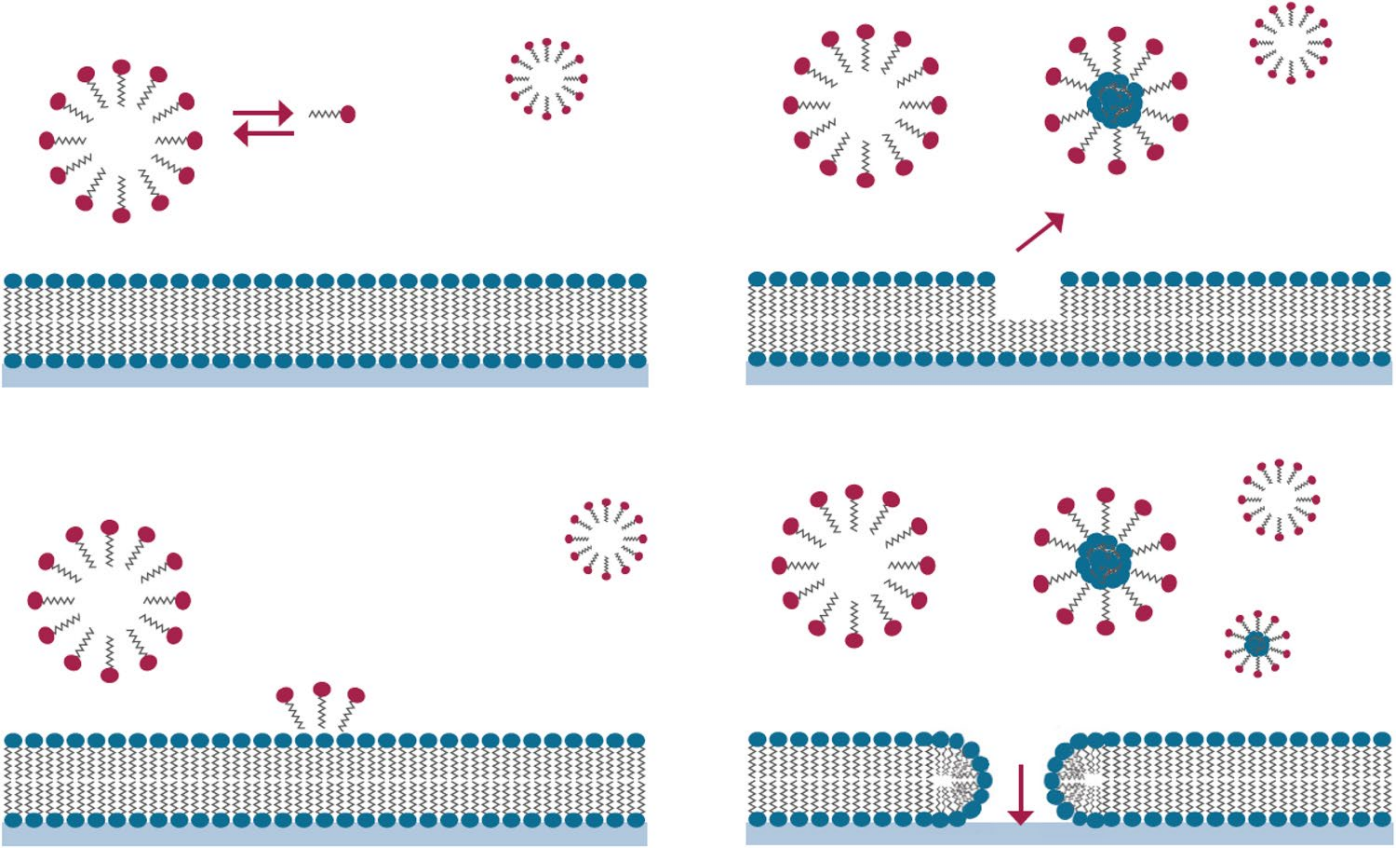
Schematic view of the effects of polidocanol on the cell membrane. The surface-active polidocanol molecules generate aggregations of molecules in the form of micelles that interact with the membrane of cells. The micelles dissolve essential molecules from the membrane, causing the affected cells to die

**Fig. 2:**
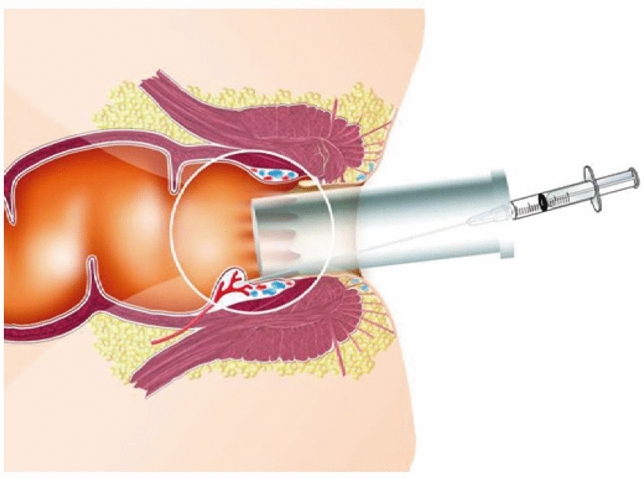
The procedure was performed in an outpatient setting with the patient in the Sims position with an open-ended anoscope and a 20G needle. 2 ml of polidocanol foam was injected above the dentate line, in each of the three classical piles

Polidocanol promotes marked vasospasm with endothelial damage and a consequent immediate inflammatory reaction within 2 min, localized sclerosis, fixation, so-called “scarring” and shrinkage of the haemorrhoidal piles due to obliteration of the vascular bed (Figs. 1 and 2).

The foam was obtained by mixing 1.6 mL of the sclerosing agent polidocanol (Aethoxysklerol^®^ 3%, Chemische Fabrik Kreussler & Co. GmbH, Wiesbaden, Germany) and 7.4 mL sterile air using the EasyFoamKit syringe system. Furthermore, the foam was re-emulsified for 20 s before each injection. A total of 2 mL of 3% polidocanol foam was used for each of the three classical piles (3, 7 and 11 o’clock).

The procedure was performed in the Sims position on an outpatient basis as previously described [12, 13] without any sedation or local anaesthesia (Fig. 2). In each centre, the same colorectal surgeon performed the procedure. The patients were asked to walk immediately after the procedure and were discharged 20 min later after a safety check.

### Outcomes

The primary outcome was the success rate after one sclerotherapy session in terms of complete resolution of bleeding episodes 1 week after the injection.

The secondary outcomes included the success rate in terms of partial or complete resolution of the symptoms at 12 months, the average number of injections necessary to resolve the bleeding, the rate of complications and adverse events (AEs), patients’ quality of life and the average time required for the return to normal daily activities, including work.

### Safety

Safety was investigated by reporting AEs, serious adverse events (SAEs) and toxicity after foam injection. Toxicity was defined using the World Health Organization (WHO) toxicity scale [14]. The AEs were classified as none, remote, possible, probable or not assessable based on the relation with the investigational drug.

### Statistical analysis

The results are reported as counts and percentages for categorical variables and as the mean ± SD (range) for continuous normally distributed variables and the median [interquartile range (IQR)] for ordinal categorical variables and for continuous nonnormally distributed variables. The chi-square test was used for crosstabulations. The time to recurrence was evaluated as the time elapsed from treatment success to the relapse of bleeding (at least 2 for the Giamundo bleeding score). Kaplan–Meier curves were used to evaluate freedom from recurrence. The changes in the HDSS, the SHS score and the Vaizey score over time were analysed with the Friedman test because these scores were not normally distributed. The results associated with a *p* value < 0.05 were considered statistically significant. Statistical data analysis was performed using IBM SPSS Statistics 20 and MedCalc 12.5.

## Results

Between January and June 2019, a total of 183 consecutive patients with 2nd-degree haemorrhoids [111 males; 60.7%, mean age 51.3 ± 13.5 (18–75) years] underwent sclerotherapy with 3% polidocanol foam in ten tertiary referral centres. One hundred and eleven patients were male (60.7%). Patient characteristics are detailed in Table 1. The mean operation time was 7.4 ± 2.5 (3–15) minutes and 23 patients experienced postoperative pain with a median VAS score of 2 (1–3). No intraoperative complications occurred. Three (1.6%) AEs (external thrombosis) were reported during the first postoperative day. These events were judged to be potentially related to the spread of the sclerosing agent below the dentate line. No SAEs occurred.

**Table 1:** Patient characteristics

Male, *N*° (%)	111/183 (60.7)
Age, years, mean ± SD (range)	51.3 ± 13.5 (18–75)
Weight (kg,mean ± SD (range)	72.8 ± 11.87 (48–101)
Height cm, mean ± SD (range)	171.82 ± 8.97 (152–190)
Body mass index, kg/m^2^, mean ± SD (range)	24.57 ± 2.9 (18.67–33.95)

**Fig. 3:**
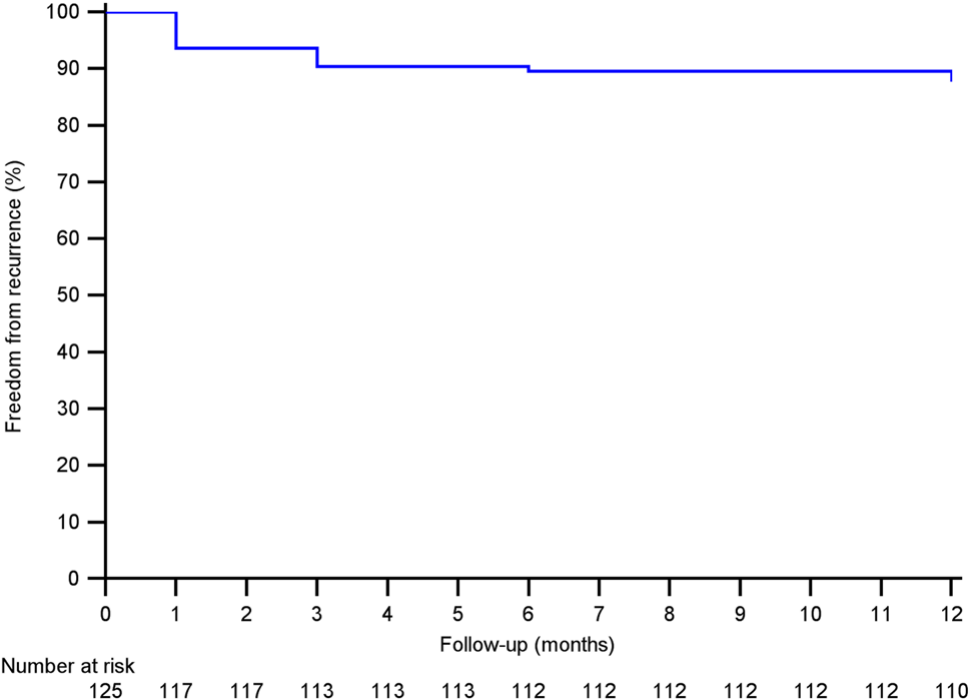
Kaplan–Meier curve concerning freedom from recurrence

Procedural results are summarized in Table 2. The primary outcome, i.e., the complete resolution of bleeding after 1 week, was reached in 125 of 183 patients (68.3%). The recurrence rate, based on the primary outcome, was 12% (15/125) (Fig. 3). Ten of 15 (66.7%) recurrences became successes at T5, 6 following a second session of sclerotherapy and 4 without performing further treatments. Two patients improved and their bleeding score stopped at 1 at the final follow-up (< 1 episode per month). Two patients underwent minimally invasive surgical treatment, i.e., dearterialization and mucopexy due to bleeding scores of 3 and 4 at T3 and T5, respectively. The last patient with recurrence underwent a third sclerotherapy session after 12 months. The patients operated on at T3 were not considered in subsequent follow-up.

**Table 2:** Procedural results

Operation time, minutes, mean ± SD (range)	7.4 ± 2.5 (3–15)
Postoperative pain (VAS > 0) *N* (%)	23/183 (12.6%)
Mean (range)	2 (1–3)
Resumption of normal activities, *N*° (%)	
0	149 (81.4)
1	28 (15.3)
2	4 (2.2)
3	2 (1.1)
Success rate after 1 week (T1) *N* (%)	125/183 (68.3)
Overall success rate (T5) *N* (%)	175/183 (95.6)
Recurrence, *N* (%)	15/125 (12)
Number of required sclerotherapy sessions for overall treatment success *n* (%)	
1	161 (92)
2	13 (7.4)
3	1 (0.6)
Adverse events, *N* (%)	
External thrombosis	3 (1.6)

*VAS* visual analogue scale

Regarding the successfully treated patients at T1, 120 of them also remained successful at T5, while 55 of 58 (94.8%) patients with a failure at T1 subsequently had a complete resolution of bleeding at T5. The overall 1-year success rate was 95.6% (175/183) (Tables 3 and 4). In particular, 13 patients (7.4%) underwent a second sclerotherapy session, while only 1 patient (1.8%) had to undergo a third sclerotherapy session.

**Table 3:** Cumulative persistence of bleeding according to Giamundo et al. [8]

	T0 (*N *= 183)	T1 (*N *= 183)	T2 (*N *= 183)	T3 (*N *= 183)	T4 (*N *= 183)	T5 (*N *= 182)
Bleeding (any frequency)	183 (100%)	58 (31.7%)	71 (38.8%)	46 (25.1%)	19 (10.4%)	7 (3.8%)

**Table 4:** Cumulative persistence of bleeding according to Giamundo et al. [8] divided by degrees of bleeding

	Preoperative (T0) (*N *= 183)	1 week (T1) (*N *= 183)	4 weeks (T2) (*N *= 183)	3 months (T3) (*N *= 183)	6 months (T4) (*N *= 182)	12 months (T5) (*N *= 182)
Bleeding (any frequency)	183 (100%)	58 (31.7%)	71 (38.8%)	46 (25.1%)	19 (10.4%)	7 (3.8%)
0	–	125 (68.3%)	112 (61.2%)	137 (74.9%)	163 (89.1%)	175 (95.6%)
1	–	50 (27.3%)	51 (27.9%)	40 (21.9%)	17 (9.3%)	4 (2.2%)
2	32 (17.5%)	7 (3.8%)	11 (6%)	–	–	–
3	92 (50.3%)	–	6 (3.3%)	5 (2.7%)	2 (1.1%)	3 (1.6%)
4	59 (32.2%)	1 (0.5%)	3 (1.6%)	1 (0.5%)	–	–

Almost all the patients (177/183; 96.7%) resumed their normal activities within 2 days of the procedure (Table 2).

The Vaizey incontinence score significantly decreased from T0 to T5 (*p *< 0.001), with 50 of 57 patients (87.7%) showing an improvement in anal continence (Table 5). This was in line with what was reported in the patient’s clinical diary, where 74 of the 81 patients (91.4%) no longer experienced soiling episodes (Table 6). A similar trend occurred for both tenesmus and itching.

**Table 5:** Vaizey score

T0	0 (0–1)
T2	0 (0–0)
T5	0 (0–0)^a^
Preoperative anal continence impairment	57/183 (31.1%)
Postoperative (12 months) improvement in anal continence	50/57 (87.7%)

^a^Median (IQR) and Friedman test (*p *< 0.001)

**Table 6:** Patients’clinical diaries

	T0 (*N *= 183)	T1 (*N *= 183)	T2 (*N *= 183)	T3 (*N *= 183)	T4 (*N *= 183)	T5 (*N *= 182)
Tenesmus						
0	78 (42.6%)	138 (75.4%)	136 (74.3%)	163 (89.1%)	163 (89.1%)	173 (94.5%)
1	84 (45.9%)	36 (19.7%)	38 (20.8%)	19 (10.4%)	18 (9.8%)	9 (4.9%)
2	21 (11.5%)	9 (4.9%)	9 (4.9%)	1 (0.5%)	1 (0.5%)	–
Itching						
0	51 (27.9%)	129 (70.5%)	128 (69.9%)	158 (86.3%)	168 (91.8%)	171 (93.4%)
1	49 (26.8%)	43 (23.5%)	43 (23.5%)	23 (12.6%)	14 (7.7%)	11 (6%)
2	50 (27.3%)	11 (6%)	12 (6.6%)	2 (1.1%)	–	–
3	33 (18.0%)	–	–	–	–	–
Soiling						
0	102 (55.7%)	163 (89.1%)	161 (88%)	168 (91.8%)	168 (91.8%)	175 (95.6%)
1	69 (37.7%)	16 (8.7%)	18 (9.8%)	13 (7.1%)	13 (7.1%)	7 (3.8%)
2	12 (6.6%)	4 (2.2%)	4 (2.2%)	2 (1.1%)	1 (0.5%)	–
Type of painkillers						
0	60 (32.8%)	154 (84.2%)	152 (83.1%)	177 (96.7%)	178 (97.3%)	178 (97.3%)
1	68 (37.2%)	26 (14.2%)	29 (15.8%)	4 (2.2%)	3 (1.6%)	4 (2.2%)
2	55 (30.1%)	3 (1.6%)	2 (1.1%)	2 (1.1%)	1 (0.5%)	–
Frequency of painkillers						
0	60 (32.8%)	154 (84.2%)	152 (83.1%)	177 (96.7%)	178 (97.8%)	178 (97.8%)
1	78 (42.6%)	20 (10.9%)	21 (11.5%)	3 (1.6%)	2 (1.1%)	3 (1.6%)
2	37 (20.2%)	8 (4.4%)	9 (4.9%)	3 (1.6%)	2 (1.1%)	1 (0.5%)
3	8 (4.4%)	1 (0.5%)	1 (0.5%)	–	–	–

**Fig. 4:**
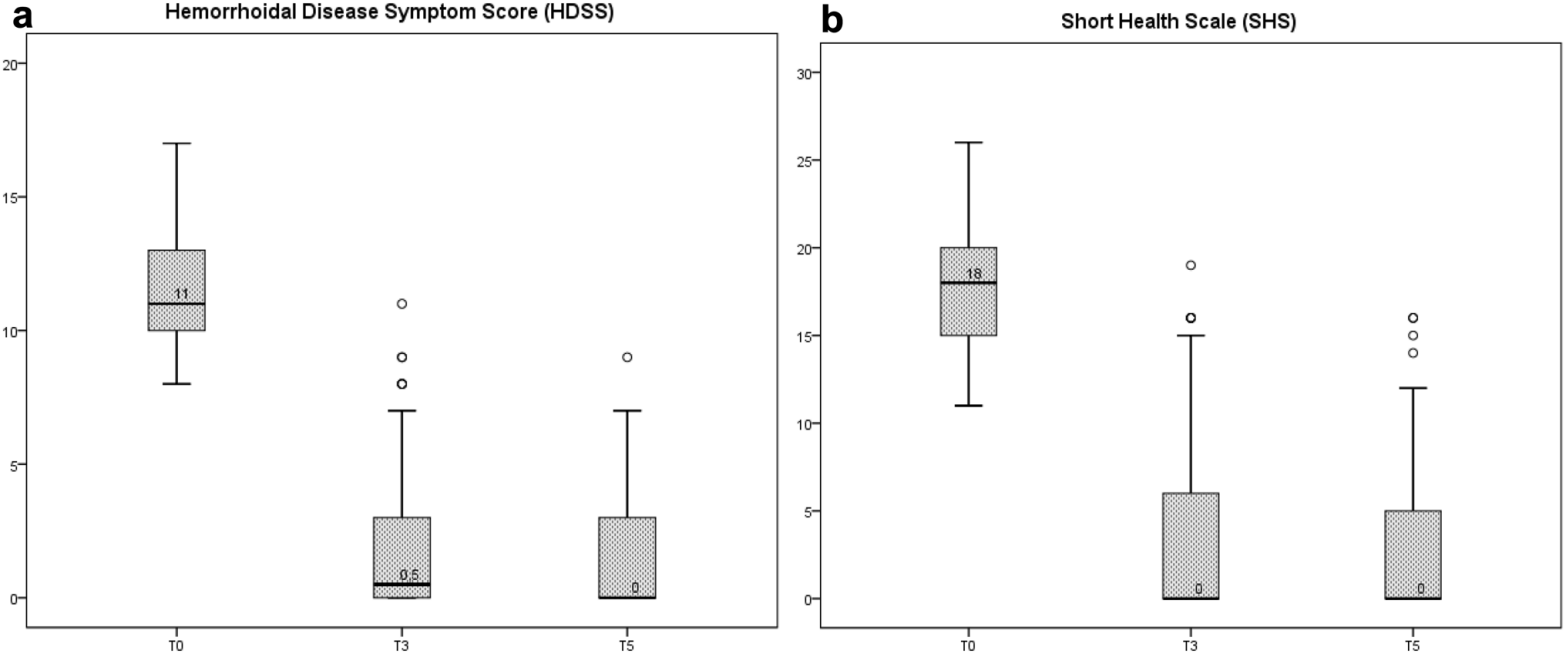
Boxplots of HDSS score (**a**) and of SHS (**b**) from T0 to T5. *HDSS* Haemorrhoidal Disease Symptom Score, *SHS* Short Health Scale

Both the HDSS and the SHS score significantly improved from a median preoperative value of 11 and 18 to 0 and 0, respectively (*p *< 0.001). Interestingly, the treatment effect, obtained by comparing the pre- and postoperative scores, showed a median change of 10 for the HDSS and of 16 for the SHS score. A total of 55.5% (101/182) of the patients were symptom-free (HDSS score = 0) after the 1-year follow-up (Tables 7 and 8), (Fig. 4).

**Table 7:** Haemorrhoidal disease symptom score and short health scale for haemorrhoidal disease

	T0	T3	T5	Treatment effect (preoperative-1 year)	*p* value
HDSS^a^	11 (10–13)	0.5 (0–3)	0 (0–3)	10 (8–12)	*p *< 0.001
SHS^a^	18 (15–20)	0 (0–6)	0 (0–5)	16 (12–19)	*p *< 0.001

Symptom-free (HDSS score = 0) at 1 year (55.5%)

^a^Median and IQR + Friedman test

**Table 8:** Mean value of the 5 symptoms of the haemorrhoidal disease symptom score

Mean value (DS) range	Pain	Itching	Bleeding	Soiling	Prolapsing
T0	2.05 (0.9) 0–4	2.3 (0.741) 0–4	3.24 (0.652) 2–4	1.74 (0.745) 0–3	2.09 (0.754) 0–4
T3	0.27 (0.592) 0–3	0.21 (0.541) 0–2	0.32 (0.678) 0–4	0.21 (0.525) 0–2	0.79 (1.017) 0–3
T5	0.25 (0.596) 0–2	0.19 (0.584) 0–3	0.02 (0.147) 0–1	0.20 (0.541) 0–2	0.74 (1.095) 0–3

On interogating the clinical diaries of the patients and the subjective evaluation of the symptoms, 79 of the 132 patients (59.8%) who had itching at T0 no longer had it at T1. Moreover, 66/81 (81.5%) and 61/105 (58.1%) patients had no more episodes of soiling and tenesmus, respectively.

There was no correlation between symptoms at T0 and recurrence. Only the presence of itching at T1 showed a significant correlation with recurrence (*p *= 0.004). In fact, 66.7% (10/15) of patients with recurrence had itching at T1, compared to only 26.4% (29/110) of patients who were free from recurrence with itching at T1. Interestingly, we observed 25.6% recurrence in patients who had itching at T0 and at T1 but only 7.8% recurrence in patients who had itching at T0 but not at T1 (*p *= 0.044).

Gender was associated with recurrence (*p *= 0.027) but not with success at T1 (*p *= 0.160) or T5 (*p *= 0.632). Indeed, 13 of the 15 patients with recurrence were men.

In general, there was no correlation between symptoms and treatment success; we observed only one significant effect for soiling (*p *= 0.012).

In fact, in patients who achieved success, soiling was present at T0 in 37.6% of patients, while in patients who had treatment failure, it was present in 58.6%.

Age, body mass index or weight did not affect either the success rate at T1 or T5 or recurrence (Table 9).

**Table 9:** Correlation with success and recurrence

	*Success at T1	*Success at T5	*Recurrences
Age	0.797	0.520	0.512
Sex	0.160	0.632	0.027
BMI	0.760	0.931	0.312
Weight	0.636	0.664	0.053
Tenesmus (T0)	0.093	0.425	0.182
Itching (T0)	1.000	0.556	0.098
Soiling (T0)	0.012	1.000	0.625
Pain (T1)	0.562	1.000	1.000

*BMI* body mass index

*p value

Despite the different number of patients enrolled there was no correlation with the success rate at T1 (*p *= 0.337) and T5 (*p *= 0.887) or recurrence (*p *= 0.634) (Table 10).

**Table 10:** Patients enrolled

Center (by affiliation)	Patients enrolled at each center
1, 13, 15	65
4	9
5	8
6	51
7	20
8	5
9	8
10	8
11	3
2, 12	6

## Discussion

In 1869, John Morgan, an Irish surgeon, was the first to describe the use of a sclerosing agent, iron sulfate, for treating HD [15]. Since then, several sclerosing agents have been used, but no real standardization has been achieved. Furthermore, the occurrence of an important number of postoperative complications has undermined the development of sclerosing treatment for HD [16].

The use of 3% polidocanol foam, however, has increased the application of sclerotherapy [17], although to date, no high-level evidence for its use in 2nd-degree haemorrhoids has been reported.

To our knowledge, this is the first and largest prospective study concerning the use of 3% polidocanol foam for 2nd-degree haemorrhoids. Furthermore, the standardization of the treatment and the use of validated scores represent the greatest strengths of this study. The immediate and consistent inflammatory reaction [13] caused by the foam was demonstrated by a success rate of 68.3% 1 week after the first injection. We do not yet know the exact durability of sclerotherapy, but many of the patients treated and not included in the 125 successes, have exhibited an improvement even without repeating the procedure. This is one of the reasons we never repeat an injection less than 4 weeks after the previous one.

Our final success rate was higher than that of Moser et al. [7], who included 1^st^-degree haemorrhoids and reported complete resolution of bleeding in 58 of 66 patients (87.9%), even without using any objective scores and with only a 12-week follow-up. Lobascio et al. [13] reported a 78.8% success rate but considered both 1st- and 2nd-degree haemorrhoids. Interestingly, using the Nystrom score [18], 78.8% of the patients were symptom-free at 1 year. In our study, 55.5% of the patients reported an HDSS of 0. The latter highlights the absence of correlation between each symptom for which bleeding has a different evolution. Interestingly, 169 of 182 patients (92.3%) had an HDSS < 5.

Recently, Salgueiro et al. [19] published the first randomized controlled trial comparing sclerotherapy with 3% polidocanol foam with rubber band ligation in one-hundred twenty patients with 1st–3rd-degree haemorrhoids.

Sclerotherapy has been shown to be superior in terms of complete success rate (88.3% versus 66.7%; *p *= 0.009), number of sessions needed to achieve the success (1.6 +  − 0.76 versus 1.3 +  − 0.60; *p *= 0.02), complications (30.0% versus 10.0%; *p *= 0.01), recurrence (41.2% versus 16.1%) and time to recurrence (10.74 months versus 11.78; *p *= 0.002). No severe complications occurred in both groups. The authors confirmed the efficacy and safety of sclerotherapy with 3% polidocanol foam even if they included 32 patients (26.7%) with 1st-degree haemorrhoids.

Interestingly, there are several differences between their technique and the technique described in the present study. Indeed, the preparation of the foam was performed with the Tessari’s method, using a higher total amount of foam and injecting it with a reusable syringe extender adapted to an intravenous needle.

Male sex has been associated with recurrence. In this context, although not statistically significant, weight showed a trend (*p *= 0.053) towards significance, probably due to the large representation of male subjects both in the recurrent group and in the whole series. Further studies will help us clarify this point.

Patients with failures at T1 had more frequent soiling at T0 than successes (58.6% vs. 37.6%).

By definition, 2nd-degree haemorrhoids prolapse but reduce spontaneously, which can be the cause of soiling. We know that this definition is not specific enough to classify HD patients; therefore, it is possible that some of these patients experience prolapse more frequently than others. The use of scoring systems helped us reduce this bias.

No SAEs occurred, demonstrating the safety and repeatability of the procedure. Our complication rate (1.6%) was consistent with a previous report by Lobascio et al. [13], in which the thrombosis rate was 1.5% (1/66 patients). This AE could be due to both the spread of the foam and the technically incorrect execution of the procedure with an injection below the dentate line.

Fifty of 57 patients with anal impairment had an improvement in the Vaizey incontinence score. Although we know that these patients did not have true faecal incontinence, it is clear that 2nd-degree haemorrhoids can temporarily prolapse with mucous discharge and paradoxical incontinence that probably resolves due to the action of scarring and fibrosis, i.e., mucopexy, induced by the foam.

Our follow-up is longer than the randomized study published by Moser et al. and consistent with the study by Salgueiro et al. [19] and Lobascio et al. [13]. Notaly, in the latter, there was no recurrence between 6 and 12 months. In our study, only 2 patients had recurrence of bleeding at T5.

Sclerotherapy is a symptomatic approach and because it obliterates vessels, its best indication is in the treatment of bleeding haemorrhoids [20, 21].

Recently, other uses of sclerotherapy have been described. In fact, the novel coronavirus disease (COVID-19) created delays in elective surgery and Lisi et al. [22] treated 10 urgent patients with 2nd–4th-degree haemorrhoids using sclerotherapy as a bridge to surgery. Last, sclerotherapy can be used in elderly patients regardless of the degree of HD, in those with or without comorbidities [6] and in patients with severe life-threatening bleeding.

The main limitation of this study is the lack of a control group. The absence of recurrences between 6 and 12 months demonstrated that our follow-up is sufficient for office-based procedures. However, the results at 3 and 5 years will clarify the effectiveness of sclerotherapy over time.

## Conclusions

Sclerotherapy with 3% polidocanol foam is a safe, promising, painless, repeatable and low-cost procedure. Its rapid action represents an arrow in the bow of any proctologist, especially in the treatment of 2nd-degree haemorrhoids. Future randomized trials with other office-based procedures, such as rubber band ligation, may confirm these results.
